# Thyroid hormone action in epidermal development and homeostasis and its implications in the pathophysiology of the skin

**DOI:** 10.1007/s40618-020-01492-2

**Published:** 2021-03-08

**Authors:** G. Mancino, C. Miro, E. Di Cicco, M. Dentice

**Affiliations:** 1 Department of Clinical Medicine and Surgery, University of Naples “Federico II”, Via S. Pansini 5, 80131 Naples, Italy; 2 CEINGE–Biotecnologie Avanzate Scarl, Naples, Italy

**Keywords:** Thyroid hormones, Thyroid hormone metabolism, Skin physiology, Deiodinases, Epithelial homeostasis

## Abstract

Thyroid hormones (THs) are key endocrine regulators of tissue development and homeostasis. They are constantly released into the bloodstream and help to regulate many cell functions. The principal products released by the follicular epithelial cells are T3 and T4. T4, which is the less active form of TH, is produced in greater amounts than T3, which is the most active form of TH. This mechanism highlights the importance of the peripheral regulation of TH levels that goes beyond the central axis. Skin, muscle, liver, bone and heart are finely regulated by TH. In particular, skin is among the target organs most influenced by TH, which is essential for skin homeostasis. Accordingly, skin diseases are associated with an altered thyroid status. Alopecia, dermatitis and vitiligo are associated with thyroiditis and alopecia and eczema are frequently correlated with the Graves’ disease. However, only in recent decades have studies started to clarify the molecular mechanisms underlying the effects of TH in epidermal homeostasis. Herein, we summarize the most frequent clinical epidermal alterations linked to thyroid diseases and review the principal mechanisms involved in TH control of keratinocyte proliferation and functional differentiation. Our aim is to define the open questions in this field that are beginning to be elucidated thanks to the advent of mouse models of altered TH metabolism and to obtain novel insights into the physiopathological consequences of TH metabolism on the skin.

## The skin organ

**Fig. 1:**
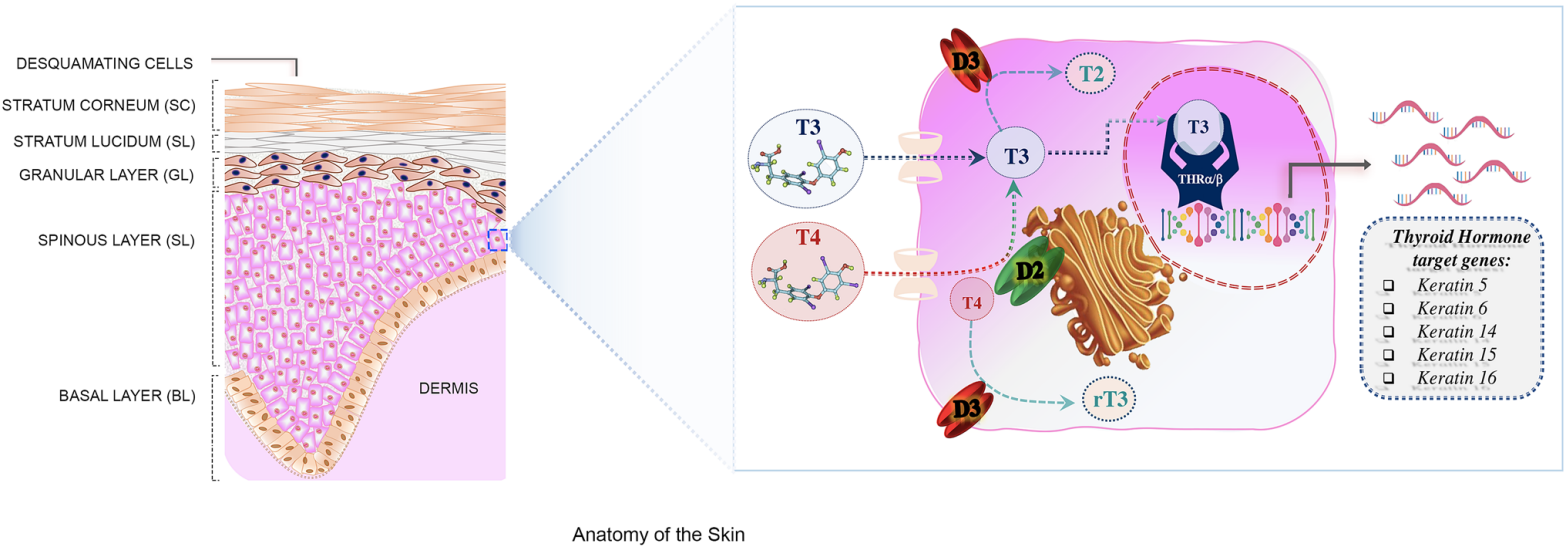
Anatomy of the skin and thyroid hormone signal. **a** The structure of the skin reflects the complexity of its functions. It is characterized by two main components, the epidermis and the dermis, which are separated by a basement membrane. The outermost level, the epidermis, is a stratified squamous epithelium, that consists of a specific constellation of cells known as “keratinocytes”, which function to synthesize keratin. The epidermis is composed of several cell layers (from inside to outside): basal layer (BL), spinous layer (SL), granular layer (GL), stratum lucidum (SL) and stratum corneum (SC). **b** The TH signal is regulated in the skin by the transport of T3 and T4 across the plasma membrane and the enzymatic activation or inactivation catalyzed by D2 and D3. Finally, the binding of T3 to TRs regulates the expression of responsive genes in the keratinocytes K5, K6, K14, K15 and K16, which confirms that TH is a key endocrine regulator that affects keratinocyte proliferation and differentiation

The skin is the largest organ of the human body and a primary interface between the “inside” and the “outside” of the body. Consequently, it is the first line of defense against physical (e.g., sun rays) and biological assaults (microbes and allergens). The skin accounts for 16% of the total body weight and thanks to its continuous self-renewal activity, it is also a metabolically active organ that participates in the maintenance of homeostasis [1, 2]. For instance, by waterproofing the most superficial cell layers, skin prevents rapid evaporation of water from the body [3]. It is also the largest sensory organ of the body. In fact, it can react to such external stimuli as heat, cold, touch and pressure and is essential to maintain temperature control [3]. Skin also plays a vital role in vitamin D production [4, 5]. The structure of skin reflects the complexity of its functions. In fact, it is divided into two main structural compartments: the outer layer (“epidermis”) and the inner layer (“dermis”) which are separated by a basement membrane that provides a stabilizing as well as a dynamic interface (Fig. 1).

The epidermis consists of four layers: the basal layer (stratum basale) above which are the spinous and granular layers and the outermost layer, the stratum corneum, which is largely responsible for the barrier function of the skin (Fig. 1). The predominant epidermal cells are the keratinocytes, which enable the epidermis to constantly self-replenish; indeed, the entire epidermis is replaced every 4 weeks [2, 4]. The bottom layer of the epidermis contains a row of undifferentiated “basal keratinocytes” that are responsible for continuous renewal of the epidermis, but only 15% of them are involved in this process, while the remaining cells are in a quiescent state. Damage to the skin triggers proliferation of quiescent cells [6]. While proliferation is restricted to the basal layer, upon differentiation basal keratinocytes move outwards through the suprabasal layers towards the surface of the skin and undergo terminal differentiation. The final product of keratinocyte differentiation is the stratum corneum, which is responsible for the barrier function of the skin against the outside environment [7, 8]. Mammalian skin is composed of pilosebaceous glands that contain a hair follicle (HF) embedded in the dermis; at the bottom of the follicle, there is the hair bulb, which is made from proliferating matrix cells and the dermal papilla (DP), which consists of specialized mesenchymal cells surrounded by the hair matrix cells. Epidermal stem cells reside in a specific region of the HF, named bulge; the epidermal stem cells ensure the continual turnover of the epidermis during skin homeostasis and regeneration. The balance between proliferation and differentiation is essential for the maintenance of the homeostasis of the skin [9].

Keratins are the main structural proteins synthesized by keratinocytes and are sequentially expressed in the layers of the epidermis [10]. The cytoskeleton of the basal keratinocytes is formed by filaments of keratin 5 and 14 (K5 and K14) [11]. Keratin gene expression changes during keratinocyte differentiation. In fact, under physiological conditions, in the spinous layer, K5 and K14 are replaced by K1 and K10 where they remain until cells are shed at the cornified layer (Fig. 1) [12]. In conditions of sustained hyperproliferation, such as psoriasis, inflammation or cancer, K1 and K10 are replaced by K6, K16 and K17 in the suprabasal layers of the epidermis [13]. Behind the epidermis and membrane basement, the dermis provides both nutritional and structural support to the epidermis. The dermis is of mesenchymal origin and the main component of the dermal matrix is the connective tissue that includes collagen IV, fibronectin and laminin. The dermis contains fibroblasts that are required for the synthesis and renewal of the extracellular matrix as well as macrophages that eliminate foreign material [14].

## Thyroid hormone action and metabolism

The concentration of TH in the bloodstream is regulated by the hypothalamic–pituitary–thyroid (HPT) axis [15]. Hypothalamic thyroid-releasing hormone (TRH) stimulates the release of thyroid-stimulating hormone (TSH) by the anterior pituitary gland. TSH stimulation in the thyroid is accompanied by the production of thyroxine (T4), which has a very low affinity for TH nuclear receptors and triiodothyronine (T3), which is the most active form of TH [16]. The biological action of TH is finely regulated by different mechanisms that mediate: TH transport across the cellular membrane, the intracellular conversion of T4 into T3 or their inactivation and the interaction of active hormone T3 with nuclear thyroid hormone receptors (TRs) and their binding to DNA. Taken together, these mechanisms culminate in the transcriptional regulation of several TH target genes [16–18]. Notably, in addition to the classical genome mechanisms of TH action, that are mediated by nuclear TRs, TH signaling occurs also by interacting with cellular proteins, such as binding with a membrane integrin αvβ3, mechanisms known as non-genomic actions of TH. In target cells, the concentration of TH is controlled by three iodothyronine deiodinase enzymes (D1, D2 and D3) that belong to the selenocysteine-containing enzyme family and share a highly conserved active site containing the selenocysteine amino acid as the key residue within their catalytic center [19] (Fig. 1). These enzymes metabolize TH through a mono-deiodination reaction that induces the removal of one iodine atom at the phenolic ring (“activation pathway”) or at the tyrosyl ring (“inactivation pathway”) of T4 and T3 [16]. In particular, D1 and D2 catalyze the conversion of T4–T3 by the removal of an iodine residue from the outer (phenolic) ring of thyroxine [20]**,** while D3 is the physiological terminator of TH activity that catalyzes the inactivation of T3 by deiodination of the inner (tyrosyl) ring of T4 to generate inactive metabolites as T2 or rT3 [21]. All three deiodinases are integral membrane proteins, but D1 and D3 are located in the plasma membrane, while D2 is found in the endoplasmic reticulum [16].

Thyroid hormone signaling in target cells results from interaction with TRs and T3–TRs complexes enhance or inhibit the expression of target genes by binding specific TH response elements (TREs) within chromatin [22]. The two isoforms of TH receptors, *Thra* and *Thrb*, are encoded by the THRA and THRB genes, respectively [21, 23]. Finally, intracellular TH action requires transport of iodothyronines across the cell membrane, a process that does not occur by passive diffusion but requires specific TH transporters [17]. Among the transporters that mediate TH influx and efflux, three have a high specificity for iodothyronines, namely OATP1C1, MCT8 and MCT10 [17].

### Effects of thyroid hormones on skin physiology

Skin is a well-established target of TH action and TH is involved in fetal epidermal differentiation, barrier formation, hair growth, wound healing, keratinocyte proliferation and keratin gene expression (24) [25]. During embryonic development, THs play a role in establishing the barrier function of the epidermis by increasing the activity of enzymes of the cholesterol sulfate cycle [26] and by affecting the development of lamellar granules [26]. Surprisingly, the expression of fully functional proteins typical of the HPT axis and in particular of the TSH and TRH receptors has been found in human skin and in HFs [27]. Receptors for TSH and TRH in the skin induce the expression of skin-specific genes [28] and thus regulate epidermal physiology, adding a mechanistic explanation of the correlation between the altered TH status and the most common dermatology diseases [29].

The action of TH on skin is mediated by TRs. Two TR isoforms have been identified in skin tissue: TR*α* and TR*β*, which act as both positive and negative regulators of transcription on various gene promoters (Fig. 1) [30]. TRs are expressed in epidermal and dermal cells, they have also been identified in skin appendages [31, 32] and both receptors can regulate, either positively or negatively, the expression of specific keratins in cultured cells [33, 34]. Studies of new born and adult human epidermal keratinocyte culture showed the conversion of T4–T3 or rT3, which suggests that deiodinases are expressed in skin [35, 36]. Subsequently, D2 was found to be expressed in human skin in vivo, in epidermal and dermal cell cultures, whereas D1 is not expressed in skin [32, 37]. D3 is not expressed at significant levels in adult peripheral tissues, while it is expressed at meaningful levels in epidermis and its expression is finely regulated during epidermal development [38–40]. It was recently demonstrated that, during mouse embryogenesis, D3 appears at E15.5 in the epidermal layers and is highly expressed at E17.5 [40]. After birth, D3 is barely detectable in the epidermal layers in early post-natal life (*P*2), it then starts to increase, reaches a peak at *P*10 and decreases thereafter [40].

Various TH target genes have been identified in skin including the keratins K5, K14, K6, K16, K15 and K17, which confirms that TH is a key endocrine regulator of keratin expression and that it affects keratinocyte proliferation and differentiation (Fig. 1) [33, 37, 41]. Thyroid hormone treatment of human skin fibroblast cultures revealed various TH-responsive genes, including a member of the RAS oncogene family (RAB3B), collagen (COLVIA3-COLVIIIA1), the hypoxia-inducible factor (HIF)-1a, a calcineurin inhibitor ZAKI 4a and members of the aldo–keto reductase (AKR) family [42]. Moreover, TH treatment induces down-regulation of alcohol dehydrogenase 1B (ADH1B) and of FGF7, which is a member of the fibroblast growth factor family that controls keratinocyte differentiation and survival [43].

During amphibian metamorphosis, skin is transformed from a bilayered non-keratinized epidermis with a thin dermis into a stratified keratinized epithelium [34]. Thyroid hormone is relevant for skin transformation and correlates with the switch of embryonic keratins to adult keratins [34]. Thyroid hormone also promotes cell proliferation of dermal fibroblasts, which is responsible for inhibition of hyaluronate synthesis resulting from down-regulation of HAS2 mediated by TH [31, 41, 44].

### Thyroid hormone and skin diseases

The epidermal pathology most frequently associated with TH alterations is systemic hyperhidrosis that affects patients with Graves’ syndrome, whereas chronic autoimmune thyroiditis, toxic multinodular goiter, toxic adenoma and exogenous TH treatment are less frequently associated with skin disorders [45]. The skin of hyperthyroid patients is dry and thinner than the skin of euthyroid subjects, as well as patients frequently experience flushing of the face, erythema of the palms and hyperhidrosis of the palms and soles (Tables 1 and 2) [46]. The skin in hypothyroid subjects, caused by Hashimoto’s thyroiditis or congenital hypothyroidism in pediatric population, appears thicker, colder and has classical myxoedema respect a euthyroid subjects (Tables 1 and 2).

**Table 1:**
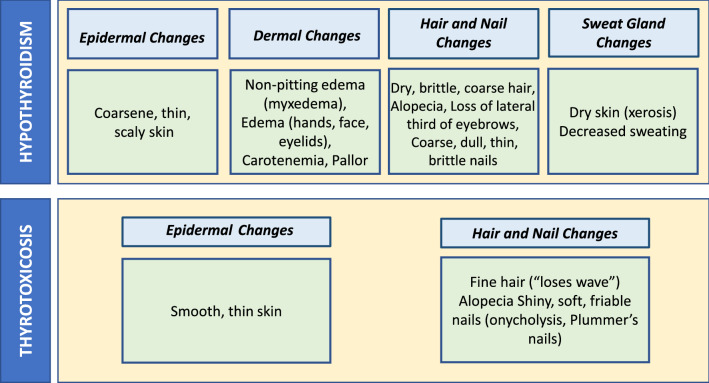
Effects of altered thyroid hormone levels on pathophysiology of the skin

**Table 2:**
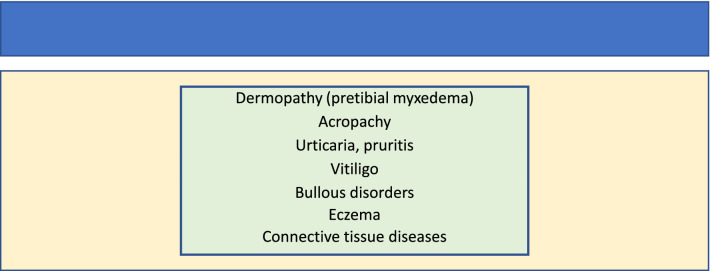
Correlation of skin disorders with chronic autoimmune thyroiditis

Most cutaneous diseases, for example vitiligo, eczema and dermatitis, are characterized by an altered immune status. This feature applies also to thyroid diseases such as chronic autoimmune thyroiditis thereby suggesting a possible interaction between these two diseases. Dermatitis herpetiformis is a rare chronic, autoimmune disease that manifests with popular vesicular rush and is associated with a variety of autoimmune diseases including Hashimoto's thyroiditis [47]. In a recent study, the prevalence of IgG class for thyroglobulin and thyroperoxidase was found to be 48% in 115 patients with dermatitis herpetiformis versus 16% in 107 unselected controls [48]. IgA class for thyroid antibodies were found in 29% of dermatitis herpetiformis patients [48]. In another study, overt thyroid disease was diagnosed in six (5%) of the dermatitis herpetiformis group and a further six patients had elevated TSH levels [48]. In addition, a study of 50 subjects suggested an association between thyrotoxicosis and dermatitis herpetiformis, on the basis of uncontrolled case reports [49]. All the afore-mentioned studies confirm the close association between epidermal diseases and the presence of thyroid autoantibodies and chronic autoimmune thyroiditis. Moreover, vitiligo, a cutaneous autoimmune disease of pigmentation, is closely associated with Hashimoto disease particularly in women with a comorbidity of up to 34% [50]. It has also been seen that in the whole sample of patients with thyroid diseases, the incidence of eczema was higher than in the control patients, mainly due to the high incidence of eczema among patients with primary thyrotoxicosis [51]. However, the mechanism underlying the association between these two diseases remains unknown. The most widely accepted hypothesis explaining the wide variety of cases in which skin diseases are associated with chronic autoimmune thyroiditis is the autoimmune hypothesis, which argues that thyroid antibodies as thyroglobulin (TgAb) and thyroperoxidase (TPOAb), are among the serum autoantibodies that react with and destroy melanocytes [48]. A fascinating hypothesis by Li et al., [52] proposes that, autoantibodies such as TgAb and TPOAb can induce a sustained oxidative stress, which in turn can result in enhanced apoptosis and senescence of melanocytes [52]. Such interesting hypothesis needs further molecular investigation.

Thyroid hormone plays an essential role in the development and maintenance of hair follicles [40], which suggests that loss of hairs may be a symptom of altered thyroid status. Alopecia is characterized by the loss of hair in patches, total loss of scalp hair (alopecia totalis) or total loss of body hair (alopecia universalis). The etiopathogenesis of hair loss is unclear, although there is evidence that autoimmunity and endocrine dysfunction may be involved [53]. Although an association between chronic autoimmune thyroiditis and alopecia has not been demonstrated, it is notable that the prevalence of thyroid disease in alopecia patients varies from 8 to 28% [54]. In one study, Kasumagić-Halilović found a significant association between alopecia and thyroid autoimmunity, as well as significantly higher antithyroid autoantibodies in alopecia patients (25.7%) than in healthy subjects (3.3%) [55].

All the studies described herein indicate a close link between altered thyroid state and skin diseases, however, the mechanism underlying this comorbidity remain unclear. Therefore, it is in the interests of the scientific community to unravel the mechanism underlying the action of TH and its alteration in epidermal homeostasis for better understanding the health of patients and improving their therapy and psychological status.

### Mouse models of impaired thyroid status in epithelial homeostasis

As noted above, the clinical evidence that hyper- and hypothyroid patients have epidermal defects such as hyperkeratosis, myxedema and sometimes alopecia, opened new questions about the link between thyroid hormones and skin homeostasis [32, 34, 56, 57]. Various mouse models of modulation of TH signaling have provided insights the molecular mechanism by which thyroid status influences skin morphology and function (see below).

#### TR mutant mice

Mouse models of thyroid hormone receptor (TR) knock out have been used to understand the role of these receptors in the skin. Most studies about the correlation between TR mutant mice and skin disorders have been performed by the group of Professor Aranda in Spain. They found that loss of TRα1 and TRβ reduces keratinocyte proliferation in the interfollicular epidermis [58]. They also found that double TRα1^−/−^β^−/−^ mice have an attenuated skin hyperplasia after 12-O-tetradecanolyphorbol-13-acetate (TPA) treatment [58]. In addition, defective proliferation in TRα1^−/−^β^−/−^ mice was associated with reduction of cyclin D1 expression and up-regulation of the cyclin-dependent kinase inhibitors p19 and p27. They also observed that these animals had increased p65/NF-B and STAT3 phosphorylation and, as a consequence, augmented expression of chemokines and proinflammatory cytokines, which demonstrates that TH and their receptors are important mediators of skin proliferation and that TRs act as endogenous inhibitors of skin inflammation [58].

In a subsequent study, Garcia-Serra et al. evaluated the role of TRs in the hair follicle cycle and in skin repair and found that TRα1^−/−^β^−/−^ mice display impaired hair cycling associated to a decrease in follicular hair cell proliferation [59] and a wound-healing defect, with retarded re-epithelialization and wound gaping associated to impaired keratinocyte proliferation [59]. The skin-phenotype of TRα1^−/−^β^−/−^ mice was not associated with the reduction in the number of bulge stem cells, responsible for hair cycling and contribute to the regeneration of the new epidermis after wounding [6]. Rather, bulge stem cell activation was reduced in the TR*α*1^−/−^*β*^−/−^ mice and was associated with aberrant activation of Smad signaling and reduced nuclear accumulation of *β*-catenin, which is crucial for stem cell proliferation and mobilization [60].

It was also demonstrated TR*α* and TR*β* depletion in mice affects the expression of several miRNAs which play a crucial role in epidermal proliferation, hair growth, wound healing and stem-cell functions [61]. In detail, the double TR*α*1^−/−^*β*^−/−^ mice were characterized by a reduction of expression levels of miR-21, miR-31, miR-34 and miR-203, with altered expression of their established targets mRNAs [61]. The functional consequence of the reduction of the expression levels of these miRNAs is suggested by the finding that many of their mRNA targets are crucial regulators of skin homeostasis, thus reinforcing the concept that TR*α*1^−/−^*β*^−/−^ mice model are useful to clarify the complex mechanism between the TH action and miRNAs in the maintenance of the skin homeostasis.

### Deiodinase mutant mice

The role of the deiodinases in the regulation of skin homeostasis and pathophysiology has mainly been investigated using conditional, epidermal-specific mice models of D2 and D3 expression (38, 62–64). To investigate the role of D3 in keratinocyte growth and differentiation, we generated an animal model for epidermal D3 loss of function. To deplete D3 in the epidermis, the Dio3^fl/fl^ mouse [65] was crossed with the transgenic mouse with the keratin 14-specific expression of a CRE recombinase, the sD3KO mouse [40]. Unlike the global D3KO mouse, in which developmental loss of D3 in the skin results in impaired clearance of TH thereby leading to elevated levels of TH action that reduce neonatal viability, growth retardation and central hypothyroidism [66], epidermal-specific D3 depletion after birth does not alter systemic THs or TSH levels [40]. However, skin physiology is altered in sD3KO mice, which highlights the importance of local regulation of TH signaling in the adult epidermis. Indeed, epidermal D3 depletion reduced skin thickness, the expression of the proliferative keratins K6 and K5 and conversely accelerated keratinocytes differentiation [40]. Moreover, sD3KO mice have a delay in skin regeneration after wound-healing damage and D3 depletion also affect hair cycle, promoting a premature catagen–telogen transition [40]. The sD3KO mouse also confirmed that TH is a key regulator of the mouse hair follicle cycle. Indeed, enhanced TH signaling results in a premature catagen–telogen transition, accompanied by an altered evolution of the hair follicle [40].

Epidermal specific depletion of D3 also resulted in enhanced tumor formation. It was recently found that D2 and D3 play a time-dependent role during skin cancer formation and progression [64]. In fact, D2 and D3 are dynamically regulated during skin tumorigenesis [64], specifically D3 is a marker of the initial stages of tumorigenesis of squamous cell carcinomas; conversely D2 expression is associated with cancer progression. Thanks to the mouse model of epithelial deiodinases-depletion, the above-mentioned sD3KO mice and K14Cre^ERT+/−^, D2 ^fl/fl^ (sD2KO) [67] enabled to assess the effective role of TH signaling in epithelial tumorigenesis. Notably, low-TH tumors (sD2KO) are fast growing tumors that have low metastatic propensity; conversely, high-TH tumors (sD3KO) grow slowly but metastasize rapidly [64]. These findings confirmed the concept that D3 is essential for the early stages of tumorigenesis [38, 63] and demonstrated that an enhanced TH signal is associated with high metastatic risk. Finally, the functional link between TH activation by D2 and keratinocyte carcinomas was recently confirmed by the finding that D2 is regulated by the transcription factor NANOG in basal cell carcinomas and squamous cell carcinomas and that D2 and NANOG expression are closely associated during the progression of keratinocyte carcinomas [68].

**Fig. 2:**
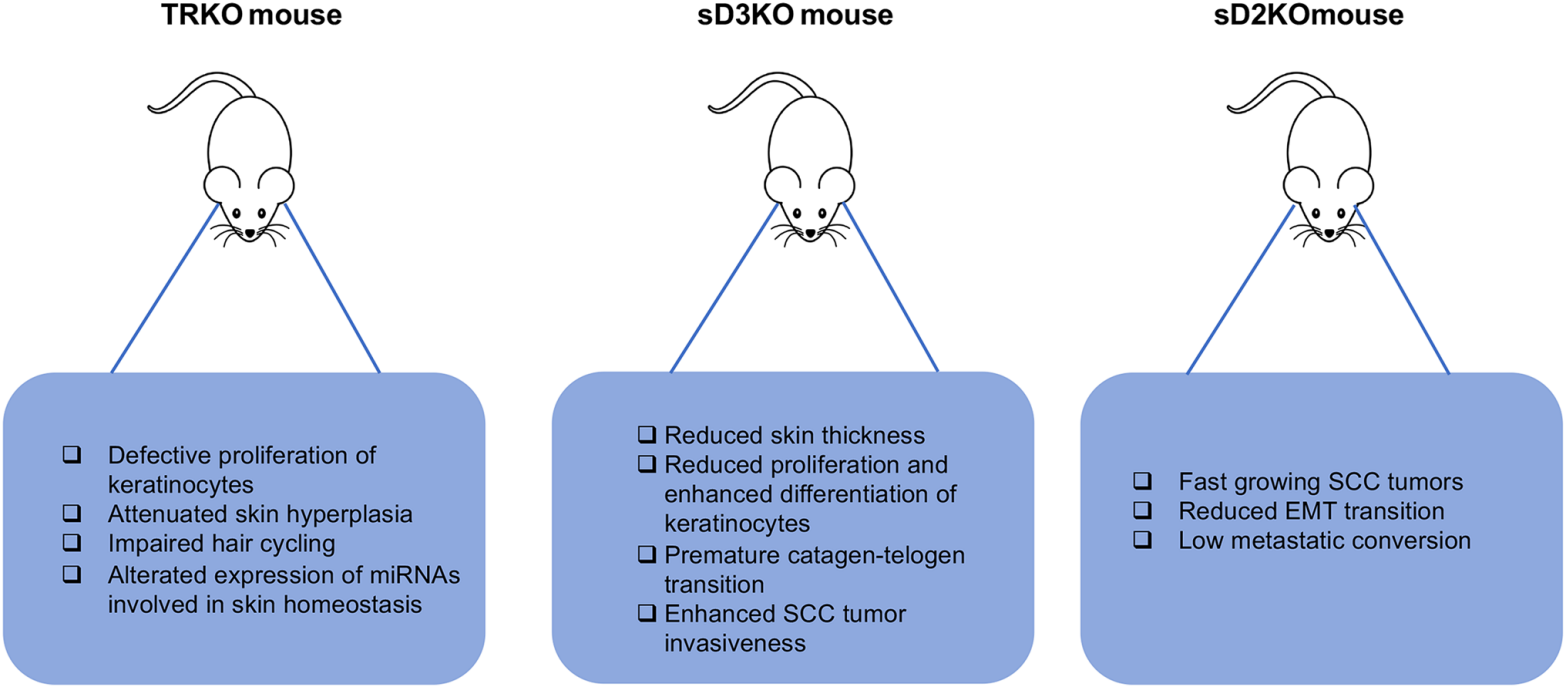
Schematic description of mouse models of TH signal alteration in the skin. TRKO mice show defective proliferation of keratinocytes in both physiological and pathological conditions. sD3KO mice show reduced proliferation and enhanced differentiation of keratinocytes and enhanced tumor invasiveness. Conversely, sD2KO mice display fasting growing Squamous Cell Carcinoma (SCC) tumors with low metastatic propensity

A comparison between TRKO mouse models and sD2KO and sD3KO mouse models reveals an apparent paradox in the role played by TH signaling in epidermal homeostasis. Indeed, while TR depletion in keratinocytes or in mouse epidermis reduces cell growth and inhibits epidermal differentiation, thereby suggesting that TH attenuation is associated with enhanced proliferation (Fig. 2), sD3KO results in reduced cell proliferation and enhanced differentiation, with the opposite occurring in the sD2KO mice, which suggests that lowering TH action results in enhanced keratinocyte cell growth. A possible explanation of the divergent phenotypes of the TR- and deiodinase-KO mice is that, in TRKO mice, the global loss of TRs can affect epidermal homeostasis starting from embryonic development, thereby reducing the TH signal in a time window in which the TH is essential for the development of several target tissues, included the epidermis. Conversely, the time-specific deiodinase depletion in the sD2KO and sD3KO mice can give insights into the specific role of TH signaling in the adult. Alternatively, the epidermal–dermal cross-talk may affect skin homeostasis. Also in this regard, while the TRKO mice are TR depleted in both the dermal and epidermal region, the sD2KO and sD3KO mice carry the loss of deiodinase expression only in the epidermal layer.

What we have learned so far from the analysis of the sD2KO and sD3KO mice models is that deregulation of TH signaling drastically influences the proportion of the basal versus suprabasal layers of the epidermis. In the case of the sD3KO mouse, this process results in a thinner epidermis with a higher proportion of differentiated keratinocytes and a reduced number of precursor cells, which could explain the skin of hyperthyroid patients, which is dry and thin [45]. The enhanced susceptibility to the inflammatory insults in both TRKO and sD3KO mice reinforces the possibility that a more pronounced tissue inflammatory response can be elicited when TH metabolism is unbalanced. This is in agreement with the association of thyroid diseases with eczema and inflammatory dermatitis [51]. Finally, since both the models of loss of TRs and loss of D3 indicate that local unbalanced levels of TH are associated with alteration of hair follicle cycle, leads to the speculation that TH is a key regulator of the precise cyclic progression of the hair follicle phases and suggest a mechanistic explanation of the association between chronic autoimmune pathologies and alopecia (54).

## Conclusions

Among their many side effects on different tissues and organs pathologies, thyroid diseases affect skin. Accordingly, decades of research led to the accumulation of multiple evidences of the influence of TH signal on epidermal development and homeostasis. However, given the complexity of array of epidermal manifestations in patients with TH disorders, much remains to be unraveled.

In the past 2 decades, in vivo analysis of mouse models of TH deregulation has contributed clarifying some aspects of the molecular mechanisms regulated by TH in keratinocytes. Although promising results have been obtained indicating how the modulation of TH signal can affect keratinocyte proliferation/differentiation balance and therefore influence pathophysiological mechanisms such as wound healing and hair follicle cycle, the final picture emerging from the available studies is still incomplete for a variety of reasons. For instance, little is known about the role of TH in epidermal development, barrier formation or epithelial-dermal cross-talk, or about the ability of TH signal alterations to influence the inflammatory response in the skin, which in turn can deeply affect the pathogenesis of skin disorders. Thus, there is a need to evaluate the role of altered TH signaling at precise stages of epidermal development, both in embryos and in TR- and deiodinase knock out models. Moreover, transcriptomic and proteomic analyses are needed to investigate the entire TH-dependent transcriptome in the skin. Unraveling how TH and its regulating molecules cooperate to regulate epidermal biology in health and disease may lead to the development of TH agonists or antagonists to treat various skin disorders.
